# Lesion Distribution and Early Changes of Right Hemisphere in Chinese Patients With Post-stroke Aphasia

**DOI:** 10.3389/fnagi.2021.632217

**Published:** 2021-12-16

**Authors:** Ruiwen Fan, Ying Gao, Hua Zhang, Xiyan Xin, Feng Sang, Zhongjian Tan, Binlong Zhang, Xiaolin Li, Xing Huang, Shuren Li, Jingling Chang

**Affiliations:** ^1^Department of Neurology, Dongzhimen Hospital, Beijing University of Chinese Medicine, Beijing, China; ^2^Key Laboratory of Encephalopathy Treatment of Chinese Medicine, State Administration of Traditional Chinese Medicine of the People’s Republic of China, Beijing, China; ^3^TCM Department of Peking University Third Hospital, Peking University, Beijing, China; ^4^State Key Laboratory of Cognitive Neuroscience and Learning, Beijing Normal University, Beijing, China; ^5^Division of Nuclear Medicine, Department of Biomedical Imaging and Image-Guided Therapy, Medical University of Vienna, Vienna, Austria

**Keywords:** aphasia, stroke, language, structural covariation, MRI, right hemisphere

## Abstract

The role of the right hemisphere (RH) in post-stroke aphasia (PSA) has not been completely understood. In general, the language alterations in PSA are normally evaluated from the perspective of the language processing models developed from Western languages such as English. However, the successful application of the models for assessing Chinese-language functions in patients with PSA has not been reported. In this study, the features of specific language-related lesion distribution and early variations of structure in RH in Chinese patients with PSA were investigated. Forty-two aphasic patients (female: 13, male: 29, mean age: 58 ± 12 years) with left hemisphere (LH) injury between 1 and 6 months after stroke were included. The morphological characteristics, both at the levels of gray matter (GM) and white matter (WM), were quantified by 3T multiparametric brain MRI. The Fridriksson et al.’s dual-stream model was used to compare language-related lesion regions. Voxel-based lesion-symptom mapping (VLSM) analysis has been performed. Our results showed that lesions in the precentral, superior frontal, middle frontal, and postcentral gyri were responsible for both the production and comprehension dysfunction of Chinese patients with PSA and were quite different from the lesions described by using the dual-stream model of Fridriksson et al. Furthermore, gray matter volume (GMV) was found significantly decreased in RH, and WM integrity was disturbed in RH after LH injury in Chinese patients with PSA. The different lesion patterns between Chinese patients with PSA and English-speaking patients with PSA may indicate that the dual-stream model of Fridriksson et al. is not suitable for the assessment of Chinese-language functions in Chinese patients with PSA in subacute phase of recovery. Moreover, decreased structural integrity in RH was found in Chinese patients with PSA.

## Introduction

Stroke is one of the leading causes of aphasia, accounting for approximately 30–40% of stroke survivors with aphasia (Engelter et al., 2006; Marebwa et al., 2017; Nouwens et al., 2018). The recovery of language function after stroke may be divided into three overlapping phases. The initial phase is called the acute phase and lasts for about 2 weeks after the onset of the stroke. The second phase is called the subacute phase, extending usually up to 6 months after the onset. The third phase, called the chronic phase, begins months to years after stroke, and it may continue for the rest of the person’s life (Hillis et al., 2006; Yang et al., 2018). The mechanisms promoting the recovery and reorganization of language in post-stroke aphasia (PSA) are still unresolved. Until now, the role of the right hemisphere (RH) in the recovery of PSA remains controversial. Previous studies suggested an important role of RH in the recovery from PSA due to left hemisphere (LH) stroke (Barlow, 1877; Weiller et al., 1995; Musso et al., 1999; Abo et al., 2004; Winhuisen et al., 2005; Turkeltaub et al., 2012). Previous studies investigating treatment-induced neural plasticity in chronic aphasia showed increased RH activation associated with better recovery in the chronic stage (Elkana et al., 2013; Kiran et al., 2015). Some studies suggest that increased RH activation may result in excessive right-to-left suppression, which may hinder language performance (Belin et al., 1996; Heiss et al., 2003; Naeser et al., 2004; Thiel et al., 2006). Moreover, the relationship between structural changes in RH and lesions in LH remains unknown. Recently, neuroimaging has been suggested to play an important role in the exploration of the implication of RH in the recovery from PSA. For a description of specific language functions related to RH structures or brain networks, local gray matter volume (GMV) is commonly measured to explain additional variance in language outcome based on T1-weighted images (Butler et al., 2014; Xing et al., 2016) along with white matter (WM) regions detected by diffusion tensor imaging (DTI) (Forkel et al., 2014; Ivanova et al., 2016; Pani et al., 2016).

Until now, several language models have been used to describe the relationship between language dysfunction and lesions in patients with PSA. The dual-stream model of language processing, one of the most used neuropsychological models of speech and language organization (Kreisler et al., 2000; Hickok and Poeppel, 2004, 2007; Fridriksson et al., 2016), has been used frequently as the classic template (Kümmerer et al., 2013; Fridriksson et al., 2016; Zündorf et al., 2016; Goucha et al., 2017; McKinnon et al., 2018; Northam et al., 2018) in order to evaluate language dysfunction concerning brain damage in PSA. This model describes two large-scale processing streams, namely, the dorsal stream is crucial for producing fluent speech and auditory-motor integration processes, and the ventral stream supports the mapping between sound and meaning and thus it is related to auditory comprehension (Saur et al., 2008; Chang et al., 2015; Sammler et al., 2015; Fridriksson et al., 2016; Garrod and Pickering, 2016). Most previous studies mainly focused on the location and extension of the brain damage after stroke, and less attention was paid to the differences in the native languages (Anglade et al., 2014). Tonal languages such as Mandarin differ from Indo-European languages in their morpho syllabic and segmental structure (Zhao et al., 2011; Leppänen et al., 2019). These differences between tonal and Indo-European languages have been evidenced not only in their linguistic features but also in their neural correlates (Wong et al., 2007) and even in genetic expression (Balter, 2006).

In this exploratory and prospective study, we investigated lesion distribution in Chinese patients with PSA in subacute phase with MRI and evaluated the language deficits from the prism of the dual-stream language model with the following purposes: (1) to study the lesion distribution and its relationship with specific language deficits in Chinese patients with PSA; (2) to explore whether the dual-stream model of Fridriksson et al. fits with the pattern of lesion deficit found in Chinese PSA and thus to conclude if the dual-stream model is valid to measure the language deficits observed in Chinese patients with PSA; and (3) to explore changes in RH of Chinese patients with PSA. To achieve these objectives, MRI analyses [e.g., voxel-based lesion-symptom mapping (VLSM), GMV analysis defined by Automated Anatomical Labeling (AAL) atlas, Tract-Based Spatial Statistics (TBSS), and tractography] were used.

## Materials and Methods

### Participants

**FIGURE 1:**
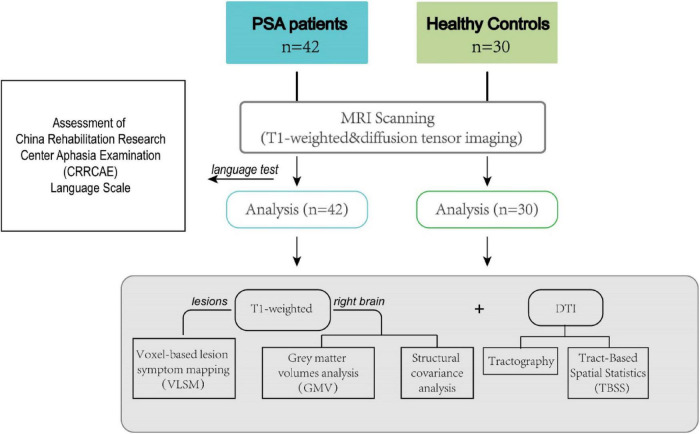
Overview of the study design.

Consecutive 42 patients (female: 13, male: 29, mean age: 58± 12) with PSA were recruited between March 2014 and June 2018 from the neurology department of Dongzhimen Hospital, Speech and Language Therapy Center of China Rehabilitation Research Centre and Neurology Department of Peking University Third Hospital. Inclusion criteria were as follows: first-ever LH ischemic stroke that happened between 1 and 6 months before language testing and MRI examination; aphasia was confirmed by a research speech-language pathologist; and all patients were right-handed based on the Edinburgh Handedness Inventory (EHI) test since the EHI test has the advantage of being a simple and brief method of evaluating laterality using a quantitative scale (Veale, 2014) and the hemispheric lateralization was also assessed by using functional MRI according to a previous publication (Wilke and Lidzba, 2007). All patients were native Chinese speakers and monolinguals. The noninclusion criteria were as follows: history of another neurological or psychiatric disease; MRI-incompatible prosthesis; and inability to perform speech and language testing as well as MRI examinations due to the severity of aphasia or comorbidity. A control group composed of 30 healthy native Chinese-speaking controls (HC) matched by age, gender, and education level were included (Figure 1). All participants have signed an informed consent form before the start of the study. This study was approved by the Institutional Review Board and Ethics Committee of Beijing University of Chinese Medicine.

### Speech and Language Testing

All patients performed a battery of language tests: Chinese Rehabilitation Research Center Standard Aphasia Examination (CRRCAE), which is designed according to the rules of Mandarin. The reliability and validity of this scale have been tested in a previous study and it has good reliability and sensitivity that can be used as a quantitative table for the diagnosis and treatment of aphasia among Mandarin users (Zhang et al., 2005; Tao et al., 2014; Zhu D. et al., 2014; Chang et al., 2017). According to the characteristics of aphasia symptoms, the scale was divided into three subitems, namely, comprehension, expression, and other speech-related abilities (e.g., calculation, copy, and cartoon description). In this study, comprehension was assessed at the reading and auditory levels, and production was measured through repetition and description levels.

### MRI Data Acquisition

Different MRI sequences were acquired in all participants to obtain GM and WM information. Neuroimaging was acquired in a Siemens 3T Trio MRI scanner (Erlangen, Germany). Axial anatomical images were acquired using a high-resolution three-dimensional T1-weighted images with the following: repetition time (TR) = 1,900 ms, echo time (TE) = 2.13 ms, time of inversion (TI) = 900 ms, flip angle = 9°, resolution = 256 × 256, and voxel size = 1.0 mm × 1.0 mm × 1.0 mm. DTI was acquired with a diffusion-weighted, single-shot, spin-echo, echo-planar imaging sequence using 30 directions with *b* = 0 s/mm^2^ and *b* = 1,000 s/mm^2^, slice thickness: 2 mm, gap = 0 mm, slices = 65, TR: 11,000 ms, TE: 94 ms, matrix: 128 × 128, field of view (FOV): 256 × 256, voxel size = 2 × 2 × 2, and phase-encoding direction: A >> P.

### MRI Data Preprocessing

#### T1-Weighted Image

T1-weighted imaging data preprocessing was carried out using SPM12^1^ and CAT12^2^ with standard processing procedures including high-dimensional DARTEL (Diffeomorphic Anatomical Registration Through Exponentiated Lie Algebra) normalization algorithms and modulation for nonlinear components. Preprocessing steps also include the segmentation of whole-brain images into GM, WM, and cerebrospinal fluid and the normalization to the DARTEL template in MNI space (Template_1_IXI555_MNI152.nii). Finally, the volume of the 90 brain GM regions defined by the AAL atlas was calculated.

#### Diffusion-Weighted Image

Diffusion-weighted imaging data were preprocessed using FMRIB Software Library (FSL^3^) (Behrens et al., 2003, 2007). First, the correction for eddy currents and subject motion was applied using the “eddy_correct” function. Then, the volume with no diffusion weighting was extracted and named “nodif.” Then, a single binarized volume in diffusion space containing ones inside the brain and zeroes outside the brain will be got by “bet2” in the “nodif.” “dtifit” fitted a diffusion tensor model at each voxel. Outputs of dtifit have mean diffusivity (MD), fractional anisotropy (FA), first eigenvalue (L1), second eigenvalue (L2), and third eigenvalue (L3). In particular, L1 is also called axial diffusivity (AD), and the mean of L1 and L2 is called radial diffusivity (RD).

The TBSS pipeline was applied with recommended parameters. Voxel-wise statistical analysis of the FA data was carried out using TBSS, part of FSL. First, the FA data of all subjects were aligned into a common space using the FMRIB nonlinear image registration tool (FNIRT), which uses a b-spline representation of the registration warp field. Next, the mean FA image was created and thinned to create a mean FA skeleton, which represents the center of all tracts common to the group. The aligned FA data of each subject were then projected onto this skeleton and the resulting data were fed into voxel-wise cross-subject statistics (Rueckert et al., 1999; Smith, 2002; Smith et al., 2004; Smith et al., 2006).

We also obtained a WM fiber bundle that crosses the GM region in which there was a significant difference between the healthy control group and the patient group. WM fiber bundles were calculated with the TrackVis toolbox.^4^ Specifically, “dti_recon” was used to build the tensor, then “dti_tracker” was for deterministic fiber tracking. The default parameters or settings were performed. The termination conditions of fiber tracking include FA value <0.2 and deflection angle >35°. We determined the WM fibers that passed through in native space, this gave fiber number (FN) maps. Referring to the abovementioned steps, the skeleton map of FN was generated.

#### Lesion Identification

**FIGURE 2:**
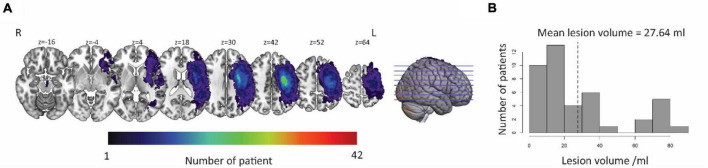
Distribution of lesion in LH. **(A)** The lesion area overlap across patients was rendered on a brain template. The color bar represents the number of patients with damage in each voxel. Numbers on top of each axial map refer to the *z*-plane of the MNI space, respectively. **(B)** Distribution of the lesion volume across the patients. The average lesion volume was 27.64 ml. L, left hemisphere; R, right hemisphere.

Lesion-symptom mapping was demarcated on T1-weighted images manually using MRIcrogl^5^ in native space by neurologists who were blinded to the language scores of participants. Figure 2 shows a lesion overlap map for the aphasic participant group. The value of each voxel represented the number of patients with damaged at this voxel. The brighter the color in the figure, the more people injured in the area.

### Statistical Analysis

#### Voxel-Based Lesion-Symptom Mapping Analysis

The purpose of VLSM analysis was used to identify the lesion area related to the language impairment. To better understand the effects of LH lesion on language performance, a VLSM was used to analyze the relationship between lesion and behavior (Bates et al., 2003) with VLSM toolbox^6^ based on MATLAB (R2016b, MathWorks, Massachusetts, MA, United States). VLSM analyses were run with 1,000 permutation tests resulting in T-maps that reflected critical regions where lesional tissue was associated with the performance on a given language measure. Significant results were derived from voxel-wise *t*-tests using a threshold of *p* < 0.05 with permutation-based correction for multiple comparisons (Lukic et al., 2017).

#### Gray Matter Volume Analysis

The GMV analysis was used to evaluate the difference in the GM areas in RH between the patient group and the healthy control group. The group differences (healthy participant group vs. aphasic patient group) in GMV were examined in all regions of RH, defined by the AAL atlas. For each region, the mean GMV was calculated. A linear regression was also used to access whether there were differences between the groups. The results were adjusted for the 45 regions with the Bonferroni correction method (corrected *p* < 0.05). In addition, we also examined the difference in the whole-brain, LH, and RH GMV between the healthy control group and the aphasic patient group.

Structural covariance between the left lesion regions and the right significant regions was assessed. Using Pearson’s correlation coefficient, the covariance between the brain regions was defined. Specifically, the co-degeneration between the volume of the brain area with a difference in the right brain and the volume of the left brain area determined by VLSM was calculated, and *p* < 0.05 was taken as the criterion for judging significance.

#### Tract-Based Spatial Statistics Analysis

The TBSS analysis was used to evaluate the difference of WM in RH between patients and healthy controls. First, we examined the relationship of FA, a measure for fiber density, axonal diameter, and myelination in WM between groups in skeleton regions of RH. A design matrix was generated using the “design_ttest2” in FSL. The FA maps were categorized as controls or patients. Statistical analysis was performed using the FSL tool “randomize” with 1,000 permutations. Threshold-Free Cluster Enhancement (TFCE; Smith and Nichols, 2009) was applied for correction of multiple comparisons. The threshold for statistical significance was *p* < 0.05. Then, the mean FA was calculated in significant skeleton regions. Other measures of WM, such as MD, AD, and RD, were also processed in the same way. Using the linear model, the relationship between the index on the WM skeleton and the language performance in the WM fiber defined in the Johns Hopkins University (JHU) template was calculated.

#### Tractography Analysis

Tractography was used to evaluate the differences of WM fibers associated with different GM regions between the patient group and the healthy control group. For tractography, the mean FN, FA, MD, AD, and RD of all subjects were calculated in the FN skeleton map. Then, we examined the differences of those indexes between the healthy control and patients with PSA by a two-sample *t*-test. In addition, the relationship between the volume of brain regions with differences in volume between the control group and the PSA group and the WM fiber indicators passing through these brain regions was calculated.

## Results

### Demographic and Behavioral Results

Patients with PSA and HC did not significantly differ in age, gender, or years of education. Stroke-related clinical characteristics of the patients were tested using the National Institutes of Health Stroke Scale (NIHSS) (Lyden et al., 2001; Cooray et al., 2015). The overall production and comprehension ability were evaluated by *z* scores of their subitems: Listen, Read, Repeat, and Speak. (The results showed that these patients had functional impairment in Listen, Read, Repeat, and Speak. Among these results, the average scores of the patients for the four tests were 16 ± 12, 16 ± 12, 10 ± 9, and 7 ± 9, out of 40, 40, 30, and 30, respectively; Table 1).

**TABLE 1:** Demographic and behavioral results.

	Total	HC	PSA	*t/*χ^2^	*p*
*n*	72	30	42	–	–
Age (years)	58 ± 12	56 ± 11	58 ± 12	64.08	0.468
Gender (F/F%)	22/31%	9/30%	13/31%	0.007	0.931
Education (years)	10 ± 4	10 ± 4	10 ± 3	−0.124	0.901
Handedness (R/L)	72/0	30/0	42/0	–	–
Time post stroke (days)	–	–	81 ± 70	–	–
Lesion volume (ml)	–	–	28 ± 25	–	–
TIV (ml)	1490 123	1483 150	1494 115	–0.354	0.725
GM volume (ml)	548 ± 61	581 ± 60	524 ± 61***	3.954	<0.001
WM volume (ml)	487 ± 54	508 ± 59	472 ± 57*	2.589	0.012
CSF volume (ml)	450 ± 89	392 ± 61	491 ± 84***	–5.497	<0.001
Left GM volume (ml)	212 ± 30	232 ± 25	198 ± 29***	5.158	<0.001
Right GM volume (ml)	221 ± 24	231 ± 24	214 ± 24**	2.899	0.005
Comprehension *Z*-score	–	–	0 ± 0.95	–	–
Production *Z*-score	–	–	0 ± 0.94	–	–
Listen	–	–	16 ± 12	–	–
Read	–	–	16 ± 12	–	–
Repeat	–	–	10 ± 9	–	–
Speak	–	–	7 ± 9	–	–
NIHSS	–	–	7 ± 4	–	–

HC, healthy control; PSA, post-stroke aphasia; TIV: total intracranial volume; GM: gray matter volume; WMV, white matter volume; CSF, cerebrospinal fluid. *p < 0.05; **p < 0.01; ***p < 0.001, versus HC.

### Voxel-Based Lesion-Symptom Mapping Results

**FIGURE 3:**
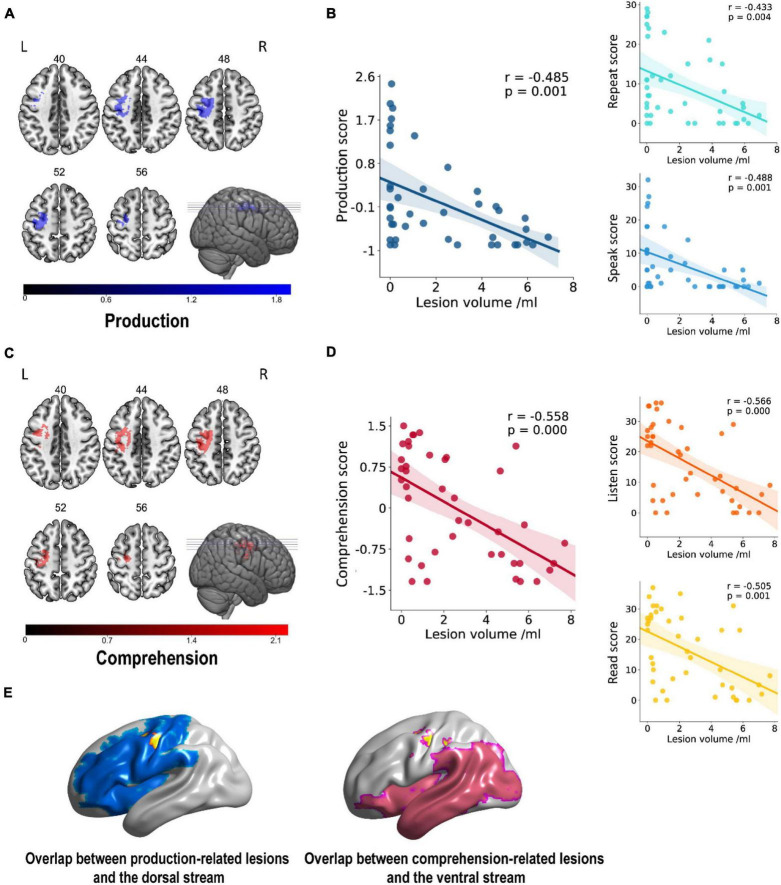
Lesion areas related to linguistic score. **(A,C)** A negative correlation was found between gray matter volume and production in the left frontoparietal cortex (cluster size = 894, peak coordinates: *x* = –28, *y* = –16, *z* = 50, *t* = 3.74, Benjamini–Hochberg correction) and between comprehension in parietal cortex and insula gyrus (cluster size = 1,046, peak coordinates: *x* = –26, *y* = –26, *z* = 48, *t* = 4.41, Benjamini–Hochberg correction). Numbers on top of each axial map refer to the *z*-plane of the MNI space. **(B,D)** Scatter plot showing partial regression using language scores as the dependent measure and gray matter extractions of cluster as the independent measure. These correlations did not differ significantly after controlling for covariates of no interest such as age, gender, the level of education, and handedness. **(E)** The overlap rates of language-related lesions (yellow), with dorsal pathway (blue) and ventral pathway (pink) of 57.72% (57.72% of all production-related lesions located in the dorsal pathway) and 0.48% (0.48% of all comprehension-related lesions located in the ventral pathway), respectively. Rate = overlapping part with dual-stream/lesion areas.

As shown in Figure 3, the comprehension- and production-related lesions were derived from VLSM analysis. There was a negative correlation between production ability and lesions in the precentral, superior frontal, middle frontal, and postcentral gyri of LH (*p* = 0.001). Similarly, the comprehension ability was negatively correlated with lesions in the precentral gyrus, superior frontal gyrus, middle frontal gyrus, insula gyrus, postcentral gyrus, and inferior parietal lobule of LH (*p* < 0.001). It is worth mentioning that the left precentral, superior frontal, middle frontal, and postcentral gyri showed a negative correlation with the performance of both comprehension and production abilities.

These resulting clusters (Table 2) were compared with areas comprised in the dorsal and ventral streams in the dual-stream model, as reported in the study by Fridriksson et al. (2016). Our results showed clear differences in lesion areas between the description of Fridriksson et al.’s dual-stream model and that of this study. The lesion areas in our results were consistent neither with the description of lesion areas in the superior frontal and middle frontal of the dorsal pathway by Fridriksson et al. nor with the lesion areas in precentral, superior frontal, middle frontal, postcentral, and inferior parietal lobule of the ventral stream (Figure 3E). Lesion overlapping for all patients is shown in Figure 3E. The overlap rates of language-related lesions (yellow) with a dorsal pathway (blue), and ventral pathway (pink) were 57.72% of all production-related lesions located in the dorsal pathway and only 0.48% of all comprehension-related lesions located in the ventral pathway.

**TABLE 2:** Overlap between lesion areas and the dual-stream model.

Region	Abbreviation	Dorsal	Ventral
Angular gyrus	ANG		Yes
Caudate nucleus	CAU		Yes
Inferior frontal gyrus, opercular part	IFGoperc	Yes	
Inferior frontal gyrus, orbital part	ORBinf	Yes	Yes
Inferior frontal gyrus, triangular part	IFGtriang	Yes	
Insula	INS	Yes	Yes (0.0048)
Middle frontal gyrus	MFG	Yes (0.0537)	
Middle occipital gyrus	MOG		Yes
Lenticular nucleus, pallidum	PAL		
Postcentral gyrus	PoCG	Yes (0.1163)	
Precentral gyrus	PreCG	Yes (0.4072)	
Lenticular nucleus, putamen	PUT	Yes	Yes
Supramarginal gyrus	SMG	Yes	Yes
Inferior temporal gyrus	ITG		Yes
Middle temporal gyrus	MTG		Yes
Temporal pole: middle temporal gyrus	TPOmid		Yes
Temporal pole: superior temporal gyrus	TPOsup		Yes
Superior temporal gyrus	STG		Yes

*The values in brackets in the dorsal and ventral columns represent the overlap rate between the VLSM result and the dual-stream model raised by Fridriksson et al. (2016).*

### Gray Matter Volume in the Right Hemisphere of Post-stroke Aphasia

**FIGURE 4:**
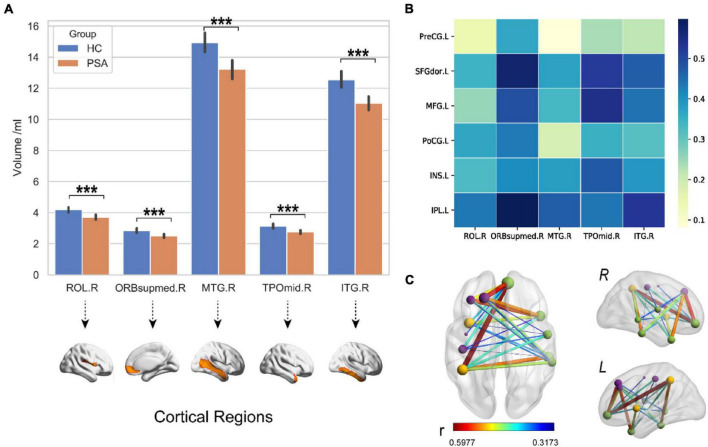
Decreased GMV in the right hemisphere. **(A)** The GMV in RH of PSA group was significantly lower than that of HC group. **(B,C)** The maps illustrate results of structural covariance analysis between language-related lesions and significantly decreased GM in RH. The color represents the size of *r* value. ROL, rolandic oper; ORBsupmed, superior frontal gyrus (medial orbital); MTG, middle temporal gyrus; TPOmid, temporal pole, middle temporal gyrus; ITG, inferior temporal gyrus; PreCG, precentral gyrus; SFGdor, superior frontal gyrus (dorsolateral); MFG, middle frontal gyrus; INS, insula; PoCG, postcentral gyrus; IPL, inferior parietal. Letters L and R in **(C)** correspond to the left and right hemispheres, respectively. ***p < 0.001.

The PSA group showed a significantly lower mean GMV than HC group (*p* < 0.001), as shown in Figure 4A. Meanwhile, a structural covariance was found between the declined GM areas and language-related lesions in LH (Figure 4B). The superior frontal gyrus, medial orbital, and middle and inferior temporal gyri were key nodes with higher weight in structural covariance (Figure 4C).

### White Matter Structure in Right Hemisphere

**FIGURE 5:**
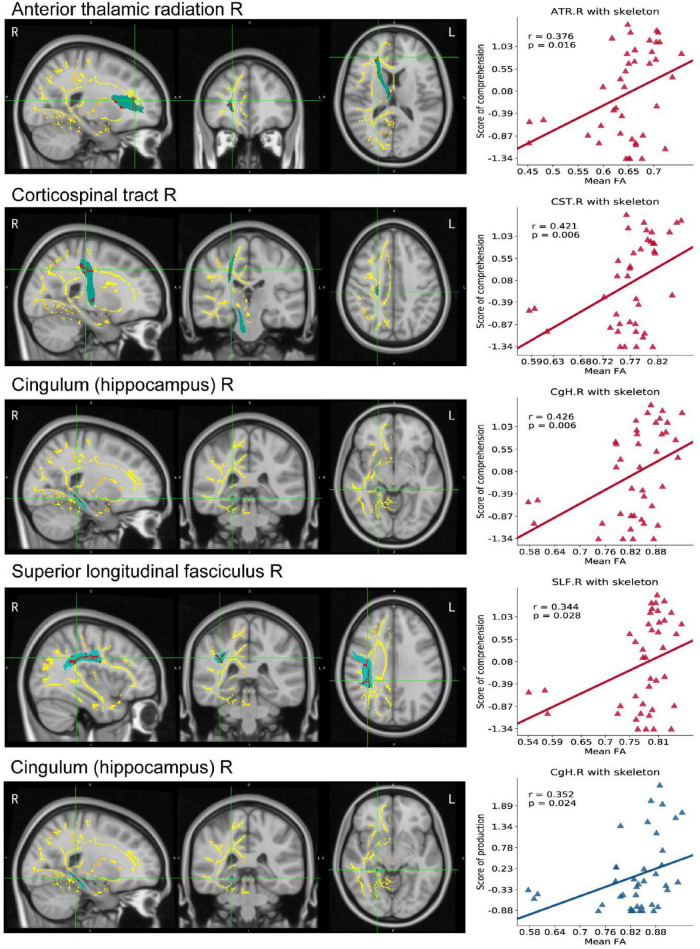
TBSS map in RH of PSA related to linguistic score. The mean FA template produced by TBSS pipeline, overlaid (axial, coronal, and sagittal view) with the skeleton generated using an FA threshold of 0.2. We do TBSS analysis in RH searching for the differences between the PSA group and the HC group (*p* < 0.05, TFCE-corrected). Red areas indicate regions with significantly reduced FA values in the PSA group compared with HC. Scatter maps represent ROI areas significantly related to the comprehension (red) or production (blue). FA, fractional anisotropy; TBSS, Tract-Based Spatial Statistics; TFCE, threshold-free cluster enhancement.

The TBSS results, as shown in Table 3, indicated lower integrity in the tracts of the PSA group as compared with those of HC. There were significant differences in FA values and MD, AD, and RD values of RH between the PSA group and the HC group. A significant decrease in FA values and a significant increase in MD, AD, and RD values were demonstrated in patients with PSA as compared with HC (Table 3). Five clusters showed a positive correlation with language abilities in patients with PSA (Figure 5).

**TABLE 3:** White matter indexes in patients with PSA compared with HC.

	HC		PSA		Test	
	Mean	SD	Mean	SD	*t*	*p*
**Indexes in skeleton by TBSS**						
FA values(×10^–1^)	7.039	0.156	6.400	0.577	5.826	<0.001
MD values(×10^–4^)	3.642	0.154	4.412	0.938	−4.387	<0.001
AD values(×10^–4^)	7.108	0.211	8.066	1.059	−4.816	<0.001
RD values(×10^–4^)	1.899	0.156	2.664	0.878	−4.649	<0.001
**Indexes in fiber by fiber tracking**						
FN	5.241	1.434	6.305	3.396	−1.592	0.116
FA values(×10^–1^)	5.330	0.220	5.070	0.574	2.320	0.023
MD values(×10^–4^)	4.285	0.172	4.703	0.846	−2.626	0.011
AD values(×10^–4^)	6.837	0.259	7.284	0.837	−2.784	0.007
RD values(×10^–4^)	2.974	0.171	3.386	0.861	−2.546	0.013

*TBSS, Tract-Based Spatial Statistics; FA, fractional anisotropy; MD, mean diffusivity; AD, axial diffusivity; RD, radial diffusivity.*

**FIGURE 6:**
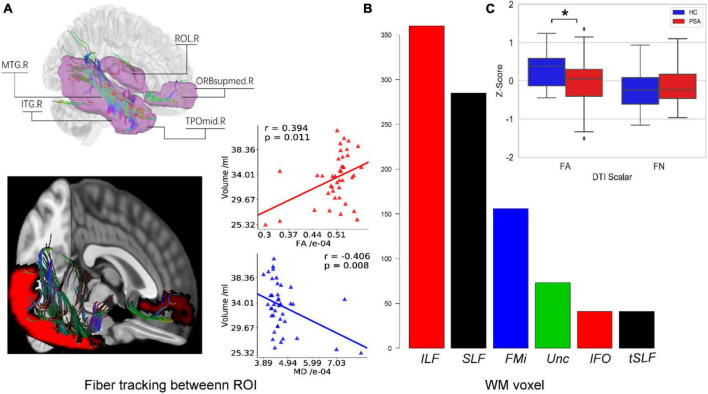
White fiber connecting reduced GM regions in RH. **(A)** All structural covariant regions (pink) in RH and WM fibers cross them are tracked by deterministic tracking. The scatter plots showed correlations between FA/MD and GMV. **(B)** The bar plot displays the voxel number of WM constructed from fiber tracing overlapped with Johns Hopkins University (JHU) WM atlas. **(C)** Box plots present differences in WM indexes between healthy people and patients. F6A is a schematic diagram of the fiber tracking. The purple area indicates the differences in the right hemisphere area between the healthy control group and the patient with PSA group. F6B represents the overlap between the fiber skeleton obtained by fiber tracking and the area defined by the WM template, which is used to determine the spatial position of the fiber. F6C represents the difference between FN and FA between the healthy control group and the patient with PSA group. ROL, rolandic oper; ORBsupmed, superior frontal gyrus (medial orbital); MTG, middle temporal gyrus; TPOmid, temporal pole middle temporal gyrus; ITG, inferior temporal gyrus; ILF, inferior longitudinal fasciculus; SLF, superior longitudinal fasciculus; FMi, forceps minor; Unc, uncinate fasciculus; IFO, inferior fronto-occipital fasciculus; tSLF, superior longitudinal fasciculus (temporal part); FA, fractional anisotropy; FN, fiber number. *p < 0.05.

Deterministic tractography analyses revealed, as shown in Figure 6A, a positive correlation between GMV and FA in the tracts passing through these areas in RH. Figures 6B,C demonstrates that reduced GMV was also associated with reduced FA in WM linking them while FN did not change significantly.

## Discussion

Our previous study has shown that the damage areas in post-stroke motor aphasia were not only located in the areas of the well-known motor speech center such as the Broca’s area, but some other damaged areas might be also involved in the formation of motor aphasia in Chinese patients with PSA (Chang et al., 2017). In this study, we found that lesions in sensorimotor areas are highly correlated with language comprehension and expression in Chinese native speakers. The present results are consistent with a previous study reporting that the human brain sensorimotor system may play an important role in the language process (Buchsbaum and D’Esposito, 2019).

Fridriksson et al. (2016) used the dual-stream model among English-speaking patients with PSA and demonstrated that distinct anatomical boundaries were revealed between a dorsal frontoparietal stream (form-to-articulation pathway) and a ventral temporal-frontal stream (form-to-meaning pathway), showing a division between two processing routes underlying English speech processing (Fridriksson et al., 2016). Previous studies performed by Ivanova et al. (2016) in native Russia-speaking patients with PSA and Rosso et al. (2015) in native French-speaking patients with PSA have shown some variabilities in the results as compared with the study results in English speakers (Hickok and Poeppel, 2004; Forkel et al., 2014), suggesting that different native languages might influence the dual-stream model although English, French, and Russian belong to a family of Indo-European languages.

Previous study (Valaki et al., 2004) has shown that tonal language such as Chinese differs from Indo-European languages not only in its written and spoken forms but also in the cerebral mechanisms involved in language functions. Valaki et al. (2004) found that while Indo-European language (e.g., Spanish and English) speakers showed strong lateralization of activity sources to LH, the Chinese demonstrated greater individual variability in the degree and direction of hemispheric asymmetries with emerging a bilaterally symmetric profile. These differences in the degree of hemispheric asymmetry were primarily due to a greater degree of activation in the right temporoparietal region in the Chinese group, suggesting increased participation of RH (Valaki et al., 2004).

In this study, lesions in sensorimotor areas correlated with both comprehension and production abilities in Chinese patients with PSA. A lesion-related language model was used in this study to distinguish the pathways of comprehension and production in native Chinese-speaking patients. The sensorimotor area and most of the frontal lobe are involved in lesion model response for both production and comprehension. We compared this model with the dual-stream model of Fridriksson et al. by calculating the overlap rate. The results showed only 0.5% of lesions related to comprehension overlap with the ventral stream, which is responsible for the meaning of language, and about 50% of lesions related to production overlap with the dorsal stream, which is responsible for expression. Furthermore, brain regions related to Chinese and Western language pathways have been reported to be different (Bolger et al., 2005; Tan et al., 2005). Previous studies have also shown that Chinese patients with PSA and English-speaking patients with PSA had different language-related GM areas (Bolger et al., 2005; Tan et al., 2005) and different WM clusters (Zhu L. et al., 2014). For the first time, to the best of our knowledge, we demonstrate in this study that Chinese patients with PSA and English-speaking patients with PSA may have similarities in production-related lesions but may be quite different in comprehension-related lesions. Our results suggested that the dual-stream model of Fridriksson et al. may not be suitable for the evaluation of the language abilities in Chinese patients with PSA in the subacute phase of recovery. Therefore, a language model should be established in the patient’s native language.

In this prospective study, a significantly structural covariance was found between reduced GMV in RH and language-related lesions in LH. Meanwhile, the TBSS skeleton of the PSA group showed decreased FA and increased MD when compared with HC groups, thus reflecting a decreased integrity of WM in RH after LH stroke. The fiber tracking through reduced GM areas also decreased in FA values with no difference in FN, indicating that the WM integrity in these areas was also affected. Previous studies have shown a compensatory increase of GMV in RH during recovery of PSA (Crinion and Price, 2005; Forkel et al., 2014; Lukic et al., 2017). However, our results demonstrated that GMV in RH significantly decreased in Chinese patients with PSA. One of the explanations for this difference may be that previous studies were done in patients who had stroke for more than 6 months before, whereas in this study, patients were included between 1 and 6 months after stroke. Another possible explanation is that there may be a difference not only in lesion distribution but also in RH structure alteration after LH stroke between Chinese patients with PSA and English-speaking patients with PSA due to different language mechanisms.

There are some limitations of this study. The first limitation is the heterogeneity in the inclusion time. Only patients in the subacute phase of recovery (between 1 and 6 months post-stroke) were included in this study. Previous study (Saur et al., 2006) has shown a difference in overall patterns of language activation for different phases after stroke. Saur et al. (2006) found a large increase of activation in the bilateral language network with the strongest increase of activation in RH language areas during the subacute phase, which was different from the pattern of language activation in the chronic phase. The imaging parameters including GMV may change during this time. Therefore, the results of patients at the beginning of subacute phase (e.g., 1-month post-stroke) may be different from those of patients 6 months post-stroke. However, most patients with PSA were included in this study between 2 and 3 months (81 ± 70 days) post-stroke; therefore, the heterogeneity of our study was relatively small. It will be interesting to know the status of GMV in RH more than 6 months after stroke in Chinese patients with PSA. Moreover, only English-speaking patients with PSA in the chronic phase (≥6 months after the onset of stroke) were included in the dual-stream model study by Fridriksson et al. (2016); therefore, this might explain partly why the dual-stream model of Fridriksson et al. may not be suitable for the evaluation of the language abilities in Chinese patients with PSA in the subacute phase of recovery. Second, Western language-speaking patients with PSA were not directly included in this study. Third, this study has shown that only a small number of patients had the lesions distributed in the ventral pathway. The muster of lesions may be typical for Chinese native speaker patients with PSA. However, further study with a larger number of patients is needed for verification of the results.

## Conclusion

In this prospective study, we investigated lesion distribution and early changes of RH in Chinese patients with PSA. Our results suggested for the first time that the dual-stream model of Fridriksson et al. is not suitable for the language evaluation of Chinese patients with PSA in the subacute phase of recovery. Furthermore, decreased GMV and low WM integrity were found in Chinese patients with PSA compared with HC.

## Data Availability Statement

The raw data supporting the conclusions of this article will be made available by the authors, without undue reservation.

## Ethics Statement

The studies involving human participants were reviewed and approved by the Institutional Review Board and Ethics Committee of Dongzhimen hospital affiliated to Beijing University of Chinese Medicine. The patients/participants provided their written informed consent to participate in this study. Written informed consent was obtained from the individual(s) for the publication of any potentially identifiable images or data included in this article.

## Author Contributions

All authors listed have made a substantial, direct, and intellectual contribution to the work, and approved it for publication.

## Conflict of Interest

The authors declare that the research was conducted in the absence of any commercial or financial relationships that could be construed as a potential conflict of interest.

## Publisher’s Note

All claims expressed in this article are solely those of the authors and do not necessarily represent those of their affiliated organizations, or those of the publisher, the editors and the reviewers. Any product that may be evaluated in this article, or claim that may be made by its manufacturer, is not guaranteed or endorsed by the publisher.

## Footnotes

1
https://www.fil.ion.ucl.ac.uk/spm/software/spm12/

2
http://www.neuro.uni-jena.de/cat/

3
http://www.fmrib.ox.ac.uk/fsl

4
http://trackvis.org/dtk/

5
http://www.cabiatl.com/mricrogl/

6
https://aphasialab.org/vlsm/
